# *Alcea rosea* L. responses to Cd and Pb stress: phenotypic, physiological, subcellular, chemical speciation, and ultrastructural analyses

**DOI:** 10.3389/fpls.2026.1778764

**Published:** 2026-03-20

**Authors:** Jianwei Liao, Yuanzhi Luo, Hongchen Yang, Wanqing Deng, Jinrong Bai, Yuanzhi Pan, Beibei Jiang, Yin Jia

**Affiliations:** 1 College of Landscape Architecture, Sichuan Agricultural University, Chengdu, China; 2 Beijing Academy of Science and Technology, Beijing, China

**Keywords:** *Alcea rosea*, cadmium, compound stress, lead, phytoremediation, tolerance

## Abstract

Cadmium (Cd) and lead (Pb) are highly toxic heavy metals that contaminate soils and aquatic environments, thereby inhibiting plant growth. Moreover, the phenomenon of combined pollution by Cd and Pb frequently occurs in natural environments. Hollyhock (*Alcea rosea*), a biennial herbaceous ornamental plant with a prolonged flowering period and abundant flowering, exhibits heightened tolerance to Cd and Pb. Although *A. rosea* is neither a hyperaccumulator nor a typical accumulator of Cd and Pb, its large biomass, strong adaptability, and high ornamental value make it a promising candidate for phytoremediation of soils with low to moderate contamination, achieving simultaneous pollution mitigation and landscape beautification. This study examined the phenotype, physiological responses, subcellular distribution, metal ion distribution and speciation, and root ultrastructure of *A. rosea* following treatment with Cd (30 μmol/L), Pb (200 μmol/L), and combined stress (Cd + Pb). Cd, Pb, and their combination inhibited the growth of *A. rosea*. The plants enhanced their tolerance against metals by increasing antioxidant enzyme activities, osmoregulatory compounds, and cellular chelators. The roots accumulated significantly more Cd and Pb than the shoots, and combined stress further raised Cd and Pb levels. Cd was primarily accumulated in the root cell walls and soluble fractions, while Pb was localized to the cell walls. The key chemical forms of Cd were FNaCl and FHAc, while FHCl and FHAc dominated for Pb. Combined stress markedly increased the contents of Cd in FE, FNaCl, FHAc, and FHCl, and Pb in FW, FHAc, and FHCl, which synergistically enhanced their toxicity and mobility (FE:80% ethanol extract; FW: double-distilled aqueous extract; FNaC1: l mol/L NaC1 extract; FHAc: 2% glacial acetic acid extract; FHCI:0.6 mol/L hydrochloric acid extract; FR: residual fraction). The ultrastructural damage included a reduction in heterochromatin, damage to the membranes, and detachment of the cell walls. This study demonstrates the potential of *A. rosea* to remediate soils co-contaminated with Cd and Pb, thereby contributing to ecological restoration efforts.

## 1 Introduction

The soil serves as an indispensable foundation for the survival and development of humans, as well as their socioeconomic progress. Soil health is directly linked to the sustainable development of human civilization. However, agricultural and industrial activities, as well as the improper disposal of waste, are the main sources of cadmium (Cd) and lead (Pb) in the soil (Mohanty and Das, 2023). It is worth noting that the combined pollution by Cd and Pb is also a significant issue, as Pb−contaminated sites are frequently contaminated with Cd as well (Huang et al., 2019). Cd and Pb ions accumulated in plants can threaten the health of humans through the food chain. Pb can cause neurological diseases (Bellinger, 2008), while low levels of Cd can induce osteoporosis, cancer, and cardiovascular diseases (Clemens et al., 2013; Satarug et al., 2010). Even low concentrations of Cd and Pb can have significant adverse effects on the growth and development of plants (Shah et al., 2010). Cd and Pb can inhibit the activity of amylase in seeds and reduce the contents of soluble sugars and reducing sugars, thereby decreasing the energy required for seed germination (Nabaei and Amooaghaie, 2019; Ou et al., 2023). Cd and Pb stress can lead to an increase in the content of reactive oxygen species (ROS) in plants, leading to oxidative damage. The effectiveness of the plant antioxidant defense system can be assessed through the activities of antioxidant enzymes, such as catalase (CAT EC 1.11.1.6), peroxidase (POD EC 1.11.1.7), and superoxide dismutase (SOD EC1.18.1.1), as well as the concentrations of non-enzymatic antioxidants, such as glutathione (GSH) and ascorbic acid (ASA) (Wani et al., 2013). Phytoremediation serves as a viable method to restore soils that have been polluted by heavy metals. Compared with physical or chemical remediation methods, it offers advantages, such as low cost, high efficiency, and minimal secondary pollution (Liu et al., 2018). In recent years, phytoremediation has been widely applied to remediate Cd and Pb pollution compared to a control group (CK), demonstrating significant ecological benefits and a high potential for application.

Plants have evolved multiple strategies to mitigate Cd and Pb toxicity. The cell wall is the first barrier to prevent Cd and Pb from entering the cells. There are many ligands on the plant cell walls that have a strong affinity for Cd and Pb. These ligands can bind to Cd and Pb through ion exchange, adsorption, complexation, and other processes and inhibit the diffusion of Cd and Pb into the plant cells, thereby alleviating their toxicity (Qin et al., 2020). Cd and Pb combine with various functional groups, such as -OH, -SH, and -COOH, attributed to the presence of carboxyl groups of pectin, which confine them to the cell wall (Nkongolo et al., 2013). In addition, plant roots reduce the toxicity to plants by chelating Cd and Pb in vacuoles. For example, Cd can combine with the sulfhydryl groups of cysteine to form non-toxic chelates (Wang et al., 2015). Phytochelatins (PCs) are a class of low molecular weight, non-protein polypeptides produced in plants that contain many sulfhydryl groups. Metallothioneins contribute to metal sequestration, homeostasis, and protection against oxidative stress (Liu et al., 2015). In addition, Yang et al. (1995) reported that the chemical forms of heavy metals are closely associated with plant tolerance. Among them, FE and FW fractions, mainly consisting of water-soluble inorganic and water-soluble organic forms, exhibit the highest mobility and toxicity. FNaCl and FHAC are mainly pectin-bound, protein-bound, and phosphate-bound forms, with relatively low mobility and toxicity. FHCl is dominated by oxalate-bound forms, which, together with FR, exhibit the lowest mobility and toxicity.

Hollyhock (*Alcea rosea* L.), a biennial erect herbaceous plant in the Malvaceae family, is a traditional Chinese flower with large and brightly colored blooms and a long flowering period; thus, it is highly valuable as an ornamental. Although *A. rosea* is not a hyperaccumulator or typical accumulator of Cd and Pb, it possesses substantial biomass, strong environmental adaptability, and high ornamental value. Consequently, it possesses the capacity for phytoremediation of soils with low to moderate contamination, thereby fulfilling both environmental remediation and landscape enhancement objectives. Moreover, *A. rosea* also demonstrates considerable tolerance to heavy metals, such as Cd and Pb (Liu et al., 2009; Wu et al., 2018). Under Cd stress, *A. rosea* exhibits enhanced tolerance through increased antioxidant activity. Under Pb stress, elevated malondialdehyde (MDA) and proline (Pro) levels, together with increased POD and CAT activities, contribute to improved Pb detoxification (Duan et al., 2022). Currently, most studies have primarily focused on the tolerance of *A. rosea* to single Cd or Pb stress, while the phenotypic, physiological responses, and tolerance of *A. rosea* to the combined stress by Cd and Pb remain unclear. We conducted experiments to examine the phenotypic and physiological changes, as well as the absorption, subcellular distribution, chemical forms, and ultrastructure of Cd and Pb in *A. rosea* under Cd, Pb, and their combination. This study aimed to explore the physiological responses and mechanisms underlying its tolerance to single and combined metal stress. This study aims to clarify how plants adapt to multiple heavy metal stresses and support future research and phytoremediation applications.

## 2 Materials and methods

### 2.1 Plant materials and experimental treatments

Seeds of *A. rosea* (a double-petaled lilac-rose cultivar planted with a plant height of 50 cm) were procured from Lanxiang Horticulture Co. (Jiuquan, China). The seeds were pretreated as follows: They were placed in tap water, and the shriveled seeds that floated on top of the water were discarded. Seeds were disinfected with 5% sodium hypochlorite for 15 min and then rinsed with distilled water. After that, they were sown in a mixed substrate of peat soil/perlite in a ratio of 4/1. After the seedlings had grown 2–3 real leaves, the soil attached to the roots was thoroughly washed. The seedlings were then transferred into 30 cm × 20 cm plastic square pots, with 24 plants allocated per pot. The plants were fixed with planting sponges, and 4.5 L of 1/8 Hoagland nutrient solution at pH = 5.8 ± 0.1 (**Supplementary Table 1**) was added for pre-cultivation. The nutrient solution was replaced once a week, and the pre-cultivation was completed after 2 weeks. The experiment was conducted in a greenhouse at Sichuan Agricultural University (30°42’ N, 103°51’ E) in Chengdu, Sichuan Province, China. The light intensity was 38 μmol/(m² s); the duration of light was 10 h, and the ambient temperature was 20–25 °C.

The concentrations of Cd and Pb that were utilized were based on the results from an unpublished preliminary experiment. Four treatments were established: (1) CK: 1/8 Hoagland nutrient solution; (2) Cd:1/8 Hoagland nutrient solution + 30 μmol/L cadmium chloride (CdCl_2_); (3) Pb:1/8 Hoagland nutrient solution + 200 μmol/L lead(II) acetate (Pb(CH_3_COO)_2_), and (4) Cd + Pb: 1/8 Hoagland nutrient solution + a combination of 30 μmol/L CdCl_2_ and 200 μmol/L Pb(CH_3_COO)_2_. A randomized complete block design was utilized with three replicates per treatment and 24 plants per treatment. The plants were harvested after 7 d of treatment to evaluate their physiological responses and tolerance.

### 2.2 Growth indicators

Growth indicators of the *A. rosea* seedlings were measured 7 d after treatment, including the plant height (the distance from the stem base to the apical meristem) and the root length (the distance from the stem base to the root tip). Shoots and roots were collected separately, and the surface debris was removed with distilled water. The seedlings were then rinsed thoroughly with deionized water and dried with a cloth. The fresh weight of each seeding was recorded. The samples were then inactivated at 105 °C for 15 min, followed by drying at 75 °C until a constant weight was achieved. The dry weight was then recorded.

### 2.3 Determination of chlorophyll content

The chlorophyll content was measured as described by Barnes et al. (1992). A total of 0.1 g of fresh leaf tissue was extracted with 10 mL of 95% acetone at 4 °C in the dark for 24 h. The absorbance was measured at 646, 663, and 450 nm using a microplate reader (SpectraMAX 190, Molecular Devices, San Jose, CA, USA), and chlorophyll content was calculated accordingly.

### 2.4 Determination of antioxidant system - related indicators

SOD (EC 1.15.1.1) was assayed as described by Giannopolitis and Ries (1977); the POD was assayed using the guaiacol method with minor modifications (Zhang et al., 2012), and the CAT was assayed by fluorophotometry as described by Zhang et al. (2015). Finally, the activities of SOD, POD, and CAT were expressed as units (U) per gram of fresh weight (FW). The content of MDA was determined as described by Heath and Packer (2022). The content of ASA (μg/g FW) was determined as described by Singh et al. (2015).

### 2.5 Determination of osmoregulatory compound contents

The contents of soluble sugars (SS) were determined as described by Heath and Packer (2022), while the content of soluble protein (SP) was determined as described by Jin et al. (2013). The content of Pro was determined using the ninhydrin method as described by Sena et al. (2024).

### 2.6 Determination of the cellular chelating compounds

Non−protein thiol (NPT) content was determined as described by Devi and Prasad, and GSH content was determined as described by Yang et al. (2018). The contents of PCs were determined by the differential method of Devi and Prasad (1998) as follows (Equation 1):

(1) PC content=content of NPT−content of GSH

### 2.7 Determination of heavy metal contents

Heavy metal concentrations were determined as described by Cheng et al. (2018), with slight modifications. Shoots were rinsed with tap and deionized water, dried with absorbent paper, and oven-dried at 80 °C to a constant weight before they were ground into powder. A total of 0.1 g (shoot) or 0.03 g (root) of each dried sample was digested overnight with nitric acid/perchloric acid (HNO_3_/HClO_4_) (5/1) in conical flasks at 280 °C on a graphene electric hotplate. After digestion, the samples were diluted to 25 mL with 1% HNO_3_, filtered, and analyzed for Cd and Pb concentrations (mg/kg) using inductively coupled plasma-mass spectrometry ICP-MS (JCELA20240031, Thermo Fisher Scientific, Bremen, Germany). CK samples were analyzed, and all treatments were analyzed in triplicate. The detection limit was 0.001 mg/L.

### 2.8 Determination of subcellular distribution of Cd and Pb

The subcellular distributions of Cd and Pb were determined as described by Lai (2015) with slight modifications. A total of 0.5 g of fresh plant roots was homogenized with pre-cooled extraction buffer and centrifuged at different speeds to obtain the cell wall (F1), organelle (F2), and soluble (F3) fractions. The fractions were digested, and the contents of Cd and Pb were determined as described in Section 2.7.

### 2.9 Determination of chemical forms of Cd and Pb in roots

Six chemical forms of Cd were sequentially extracted from the roots as described by Wu et al. (2005). These included the ethanol-extractable fraction (FE), water-extractable fraction (FW), sodium chloride-extractable fraction (FNaCl), acetic acid-extractable fraction (FHAc), hydrochloric acid-extractable fraction (FHCl), and residual fraction (FR). The tissues were digested as described above, and the chemical forms of Cd and Pb were quantified.

### 2.10 Observation of root ultrastructure

The roots of *A. rosea* were longitudinally sectioned and then fixed with glutaraldehyde (OHC(CH_2_)_3_CHO). The sections were then post-fixed in osmic acid. The fixed samples were dehydrated in an ethanol series of increasing concentrations until 100% and ultimately embedded in Epon812 epoxy resin. The ultrathin sections were sliced using a microtome (LKB, Paris, France), and observed and photographed on a Hitachi HT7800 transmission electron microscope (Hitachi High-Tech Corporation, Tokyo, Japan) at 80 kV.

### 2.11 Statistical analysis

All data represent the average of three independent biological experiments and are expressed as the mean ± SD. Statistical analyses were performed using SPSS 26.0 (IBM, Inc., Armonk, NY, USA), including a one-way analysis of variance (ANOVA), independent samples t-tests, correlation analysis, and principal component analysis (PCA). The graphs were drawn using Adobe Photoshop 2020 (Adobe, San Jose, CA, USA) and Origin 2024 (OriginLab, Northampton, MA, USA).

## 3 Results

### 3.1 Growth parameters

**Figure 1:**
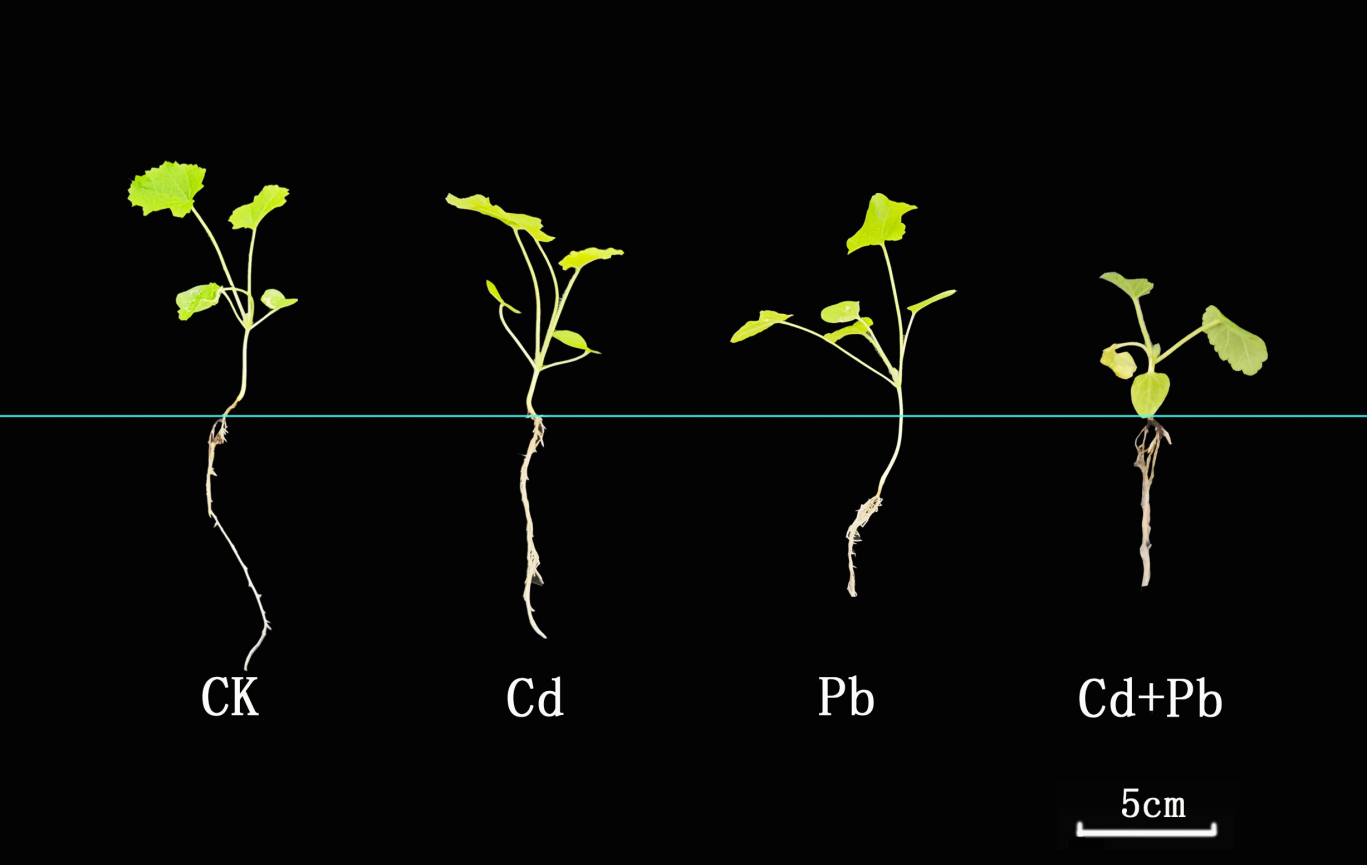
Effects of Cd, Pb and their combined stress on the plant morphology of *A. rosea*.

Compared to CK, the Pb and Cd + Pb treatments significantly reduced the height of *A. rosea* plants, which decreased by 6.7% and 42.7%, respectively (*p<* 0.05). All three metal treatments (Cd, Pb, and Cd + Pb) markedly reduced root length, with decreases of 14.3%, 40.7% and 50.6% (*p<* 0.05), respectively (**Table 1**; **Figure 1**). Relative to the CK group, the fresh weight of the shoot tissue in the Pb and Cd + Pb groups decreased markedly by 38.4% and 57.2%, respectively (*p<* 0.05). In terms of the root fresh weight, the Cd, Pb, and Cd + Pb treatments all significantly reduced root fresh weight (*p<* 0.05), with decreases of 28.0%, 42.7%, and 62.2%, respectively. There were no significant differences in shoot dry weight among the Cd, Pb, and Cd+Pb treatments compared with the CK group (*p >* 0.05). The Pb treatment group had a noticeably greater root dry weight than the CK group, with an increase of 50.0%, while there was no significant change (*p >* 0.05) in the Cd and Cd + Pb treatment groups (**Table 1**).

**Table 1:** Growth indicators of *A. rosea* under Cd, Pb and their combined stress.

Parameter	Plant height (cm)	Root length (cm)	Shoot fresh weight (g)	Root fresh weight (g)	Shoot dry weight (g/5 plants)	Root dry weight (g/5 plants)
CK	8.9 ± 0.451a	9.1 ± 0.265a	0.388 ± 0.048a	0.082 ± 0.019a	0.133 ± 0.023ab	0.016 ± 0.003b
Cd	8.6 ± 0.100ab	7.8 ± 0.436b	0.444 ± 0.071a	0.059 ± 0.005b	0.155 ± 0.026a	0.019 ± 0.002ab
Pb	8.2 ± 0.231b	5.4 ± 0.231c	0.239 ± 0.055b	0.047 ± 0.007bc	0.122 ± 0.015ab	0.024 ± 0.003a
Cd + Pb	5.1 ± 0.346c	4.5 ± 0.635d	0.166 ± 0.012b	0.031 ± 0.005c	0.093 ± 0.011b	0.017 ± 0.005ab

Values are means ± SD (n = 3). Values with a different letter within a sampling date are significantly different (*p* < 0.05).

### 3.2 Photosynthetic pigment content

**Figure 2:**
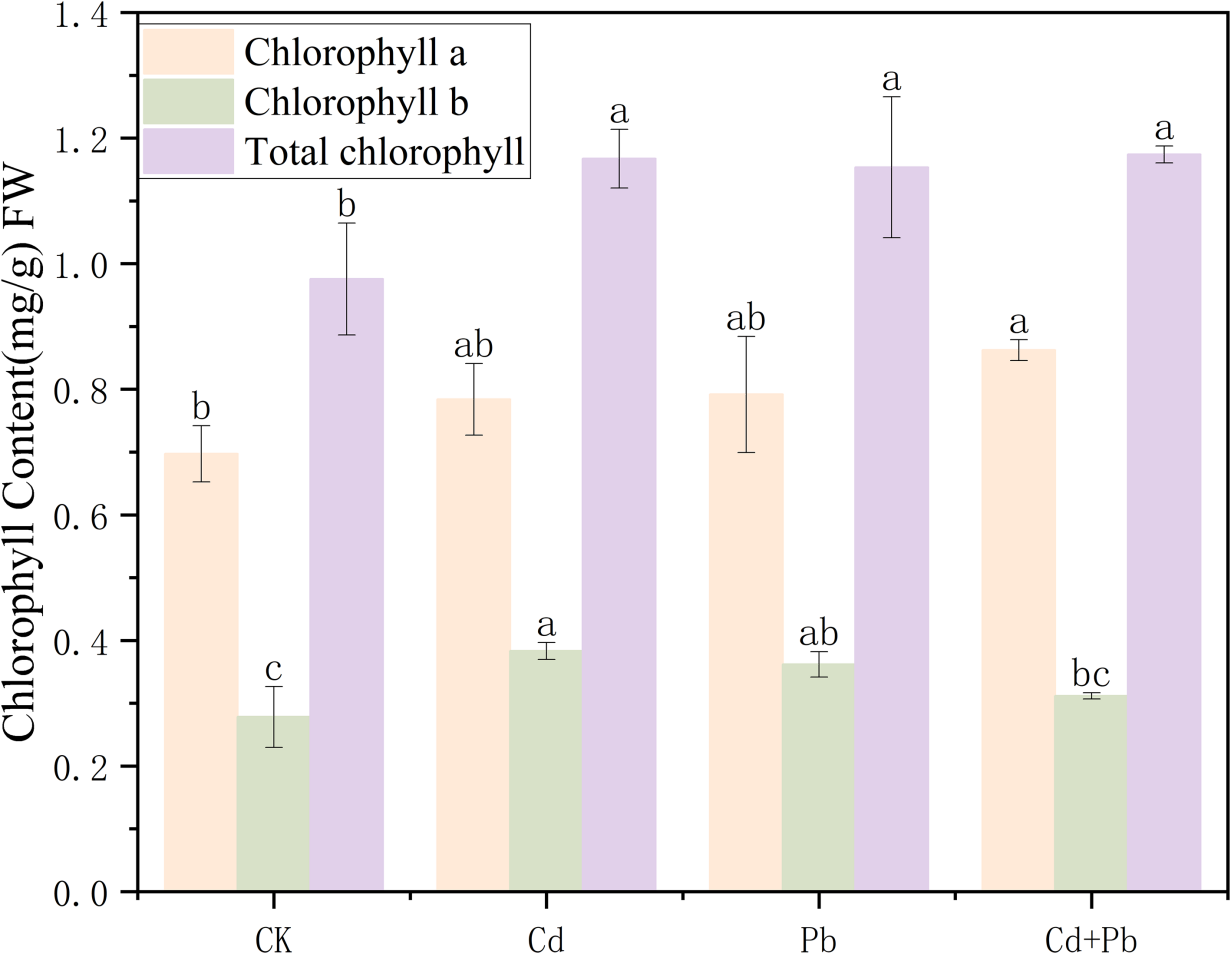
Effects of Cd, Pb and their combined stress on chlorophyll content of *A. rosea.* FW indicates fresh weight. Values are means ± SD (n = 3). Values with a different letter within a sampling date are significantly different (*p* < 0.05). FW indicates fresh weight.

In comparison to the CK group, the Cd, Pb, and Cd + Pb treatments all significantly increased the content of chlorophyll a in *A. rosea* (*p<* 0.05), with increases of 12.4%, 13.5%, and 19.1%, respectively. The Cd and Pb treatments markedly increased the content of chlorophyll b by 37.7% and 30.1% (*p<* 0.05), respectively, whereas no significant difference was observed in the Cd + Pb group compared with the CK (*p >* 0.05). The total chlorophyll contents in the Cd, Pb, and Cd + Pb groups were all substantially higher than those in the CK group (*p<* 0.05), with increases of 19.6%, 18.4%, and 20.3%, respectively (**Figure 2**).

### 3.3 Antioxidant enzyme activities and contents of MDA and ASA

**Figure 3:**
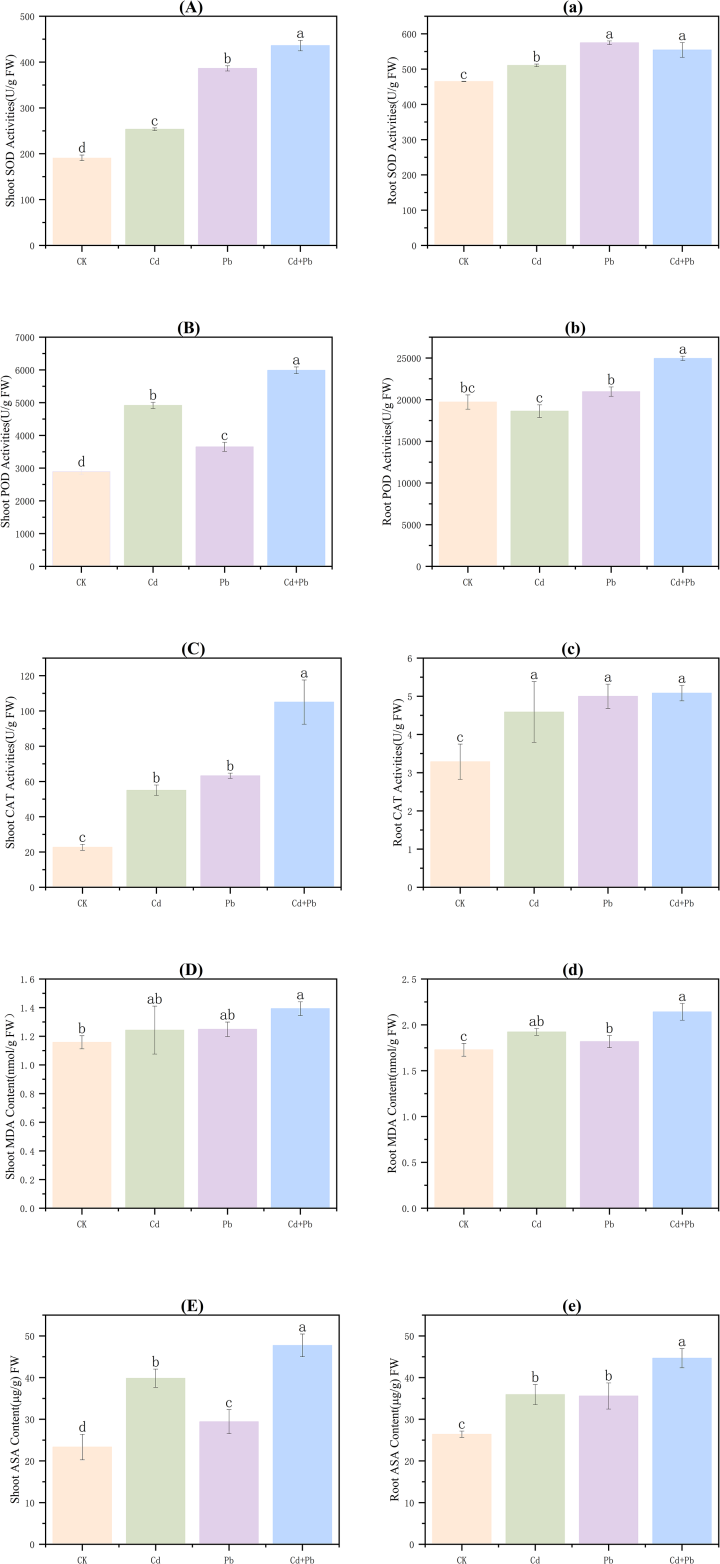
Effects of Cd, Pb and their combined stress on antioxidant enzyme activities, MDA and ASA content of *A. rosea.* Capital letters **(A–E)** represent the antioxidant enzyme activities, MDA and ASA content in the shoots, and lowercase letters **(a–e)** represent the antioxidant enzyme activities, MDA and ASA content in the roots. FW indicates fresh weight. Values are means ± SD (n = 3). Values with a different letter within a sampling date are significantly different (*p* < 0.05).

Relative to CK, SOD, POD, and CAT activities, as well as MDA and ASA contents, increased significantly in the shoots under all metal treatments (*p<* 0.05). The activity of SOD increased by 32.7%, 102.3% and 128.4%, respectively (**Figure 3A**), and that of POD was 1.7-, 1.3-, and 2.1-fold more than that of the CK, respectively (**Figure 3B**). The activity of CAT was 2.4-, 2.8-, and 4.6-fold greater than that of the CK, respectively (**Figure 3C**). The content of MDA increased by 7.3%, 7.9% and 16.9%, respectively (**Figure 3D**). The ASA content was 1.7-, 1.3-, and 2.0-fold greater than that of the CK, respectively (**Figure 3E**). In addition, relative to the Cd group and Pb group, the activity of SOD in the Cd + Pb group increased substantially by 72.1% and 12.9% (*p<* 0.05), respectively (**Figure 3A**), while CAT activity increased by 90.7% and 66.0% (*p<* 0.05), respectively (**Figure 3C**).

In the roots, in comparison to the CK, SOD activity in the Cd, Pb and Cd + Pb groups increased markedly by 9.8%, 23.6% and 19.2% (*p<* 0.05), respectively (**Figure 3a**). POD activity did not differ significantly under Cd or Pb alone (*p >* 0.05), but increased by 26.6% under Cd + Pb (**Figure 3b**). The activity of CAT increased by 39.7% in the Cd group, 52.1% in the Pb group, and 54.7% in the Cd + Pb group (**Figure 3c**). Moreover, MDA content increased by 11.3%, 5.2%, and 24.0%, respectively (**Figure 3d**). In addition, relative to the CK, the contents of ASA in the Cd, Pb and Cd + Pb groups increased considerably by 70.7%, 26.0% and 69.0% (*p<* 0.05), respectively (**Figure 3e**).

### 3.4 Osmoregulatory compound content

**Figure 4:**
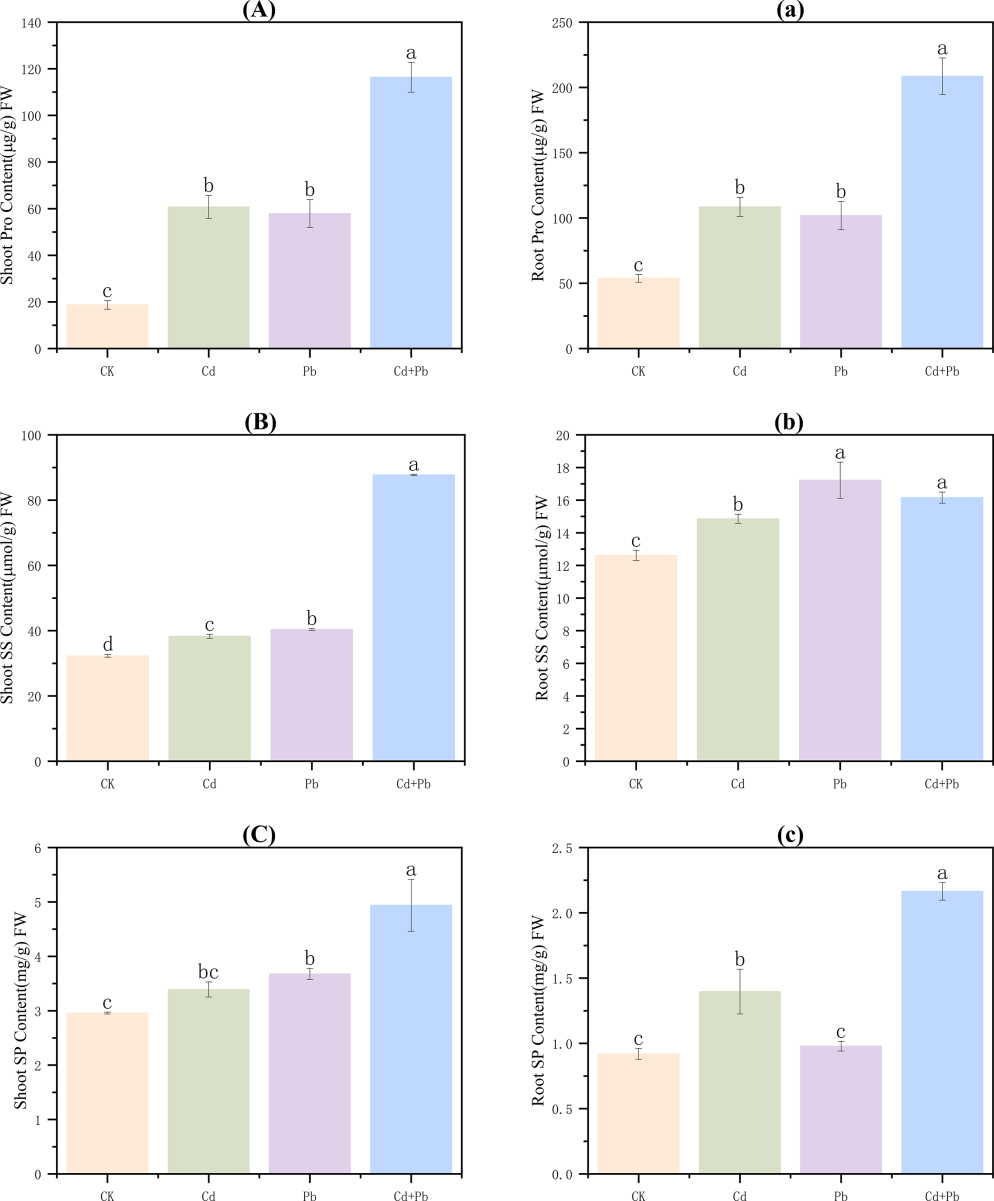
Effects of Cd, Pb and their combined stress on the contents of osmotic adjustment substances in *A. rosea.* Capital letters **(A–C)** represent the contents of osmotic adjustment substances in the shoots, and lowercase letters **(a–c)** represent the contents of osmotic adjustment substances in the roots. FW indicates fresh weight. Values are means ± SD (n = 3). Values with a different letter within a sampling date are significantly different (*p* < 0.05).

Proline (Pro), soluble sugars (SS), and soluble protein (SP) contents in shoots increased significantly under all metal treatments compared with the CK (*p<* 0.05). The content of Pro was 3.2-, 3.1-, and 6.2-fold higher than that of the CK under Cd, Pb, and Cd + Pb, respectively (**Figure 4A**). The contents of SS increased by 18.5%, 24.9% and 171.5%, respectively (**Figure 4B**), while SP increased by 14.7%, 24.3% and 135.3% (**Figure 4C**). Relative to the Cd group, the Cd + Pb group was 91.7% and 129.1% higher than the contents of Pro and SS, respectively. When compared with the Pb group, these values were 101.1% and 117.3% higher (**Figures 4A, B**).

In the roots, in comparison to the CK, the contents of Pro and SS in the Cd, Pb, and Cd + Pb groups were markedly increased (*p<* 0.05). Pro increased by 102.0%, 89.6% and 288.2%, respectively (**Figure 4a**). SS increased by 17.7%, 36.5% and 28.0%, respectively (**Figure 4b**). SP content did not differ significantly in roots under Pb (*p* > 0.05), but increased 1.5− and 1.7−fold under Cd and Cd + Pb, respectively (**Figure 4c**). Compared with the Cd group and Pb group, the content of Pro in the Cd + Pb group increased significantly by 92.2% and 104.8% (*p<* 0.05), respectively (**Figure 4a**).

### 3.5 Content of cellular chelating substances

**Figure 5:**
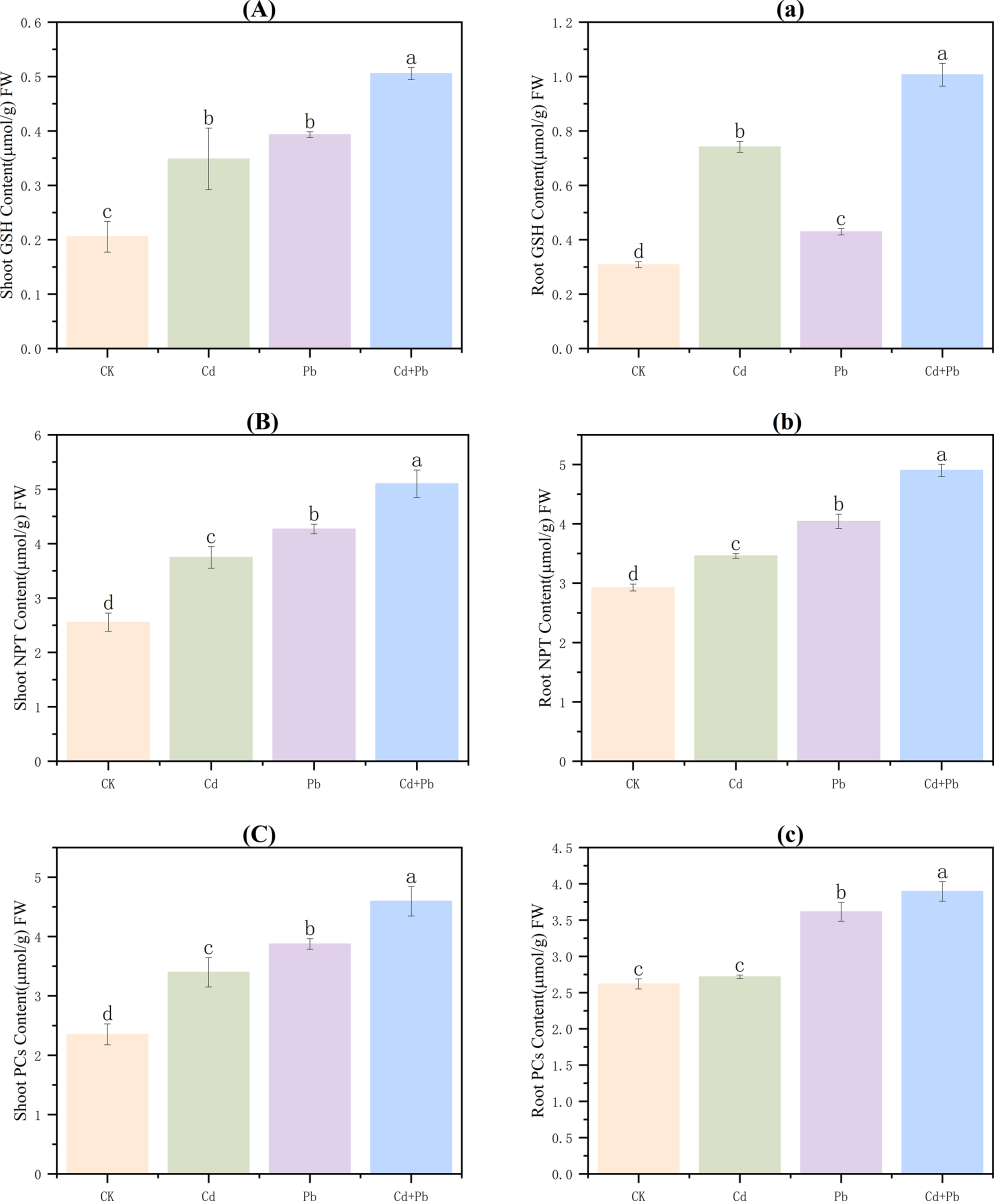
Effects of Cd, Pb and their combined stress on the contents of cellular chelating substances in *A. rosea.* Capital letters **(A–C)** represent the contents of cellular chelating substances in the shoots, and lowercase letters **(a–c)** represent the contents of cellular chelating substances in the roots. FW indicates fresh weight. Values are means ± SD (n = 3). Values with a different letter within a sampling date are significantly different (*p<* 0.05).

In the shoots, compared with the CK, the contents of GSH, NPT, and PCs in the Cd, Pb, and Cd + Pb groups all increased significantly (*p<* 0.05). GSH contents were 1.7-, 1.9-, and 2.5-fold higher than that of those CK, respectively (**Figure 5A**). NPT increased by 46.6%, 67.0% and 99.5%, respectively (**Figure 5B**), while the contents of the PCs increased by 44.6%, 64.9% and 95.4%, respectively (**Figure 5C**). Compared with Cd and Pb alone, the Cd + Pb treatment increased GSH by 45.7% and 34.4%, and NPT by 36.1% and 19.5%, respectively (**Figures 5A, B**).

In the roots, relative to CK, NPT, and GSH contents increased significantly across the Cd, Pb, and Cd + Pb groups (*p<* 0.05). GSH increased by 140.7%, 39.4% and 226.9%, respectively (**Figure 5a**). NPT increased by 18.2%, 38.1% and 67.5%, respectively (**Figure 5b**). PC content did not differ significantly under Cd (*p* > 0.05), but increased 1.4− and 1.5−fold under Pb and Cd + Pb, respectively (**Figure 5c**). Compared with Cd and Pb alone, GSH content in the Cd + Pb group increased by 35.8% and 134.4% (*p<* 0.05), respectively (**Figure 5a**).

### 3.6 Contents of Cd and Pb

**Figure 6:**
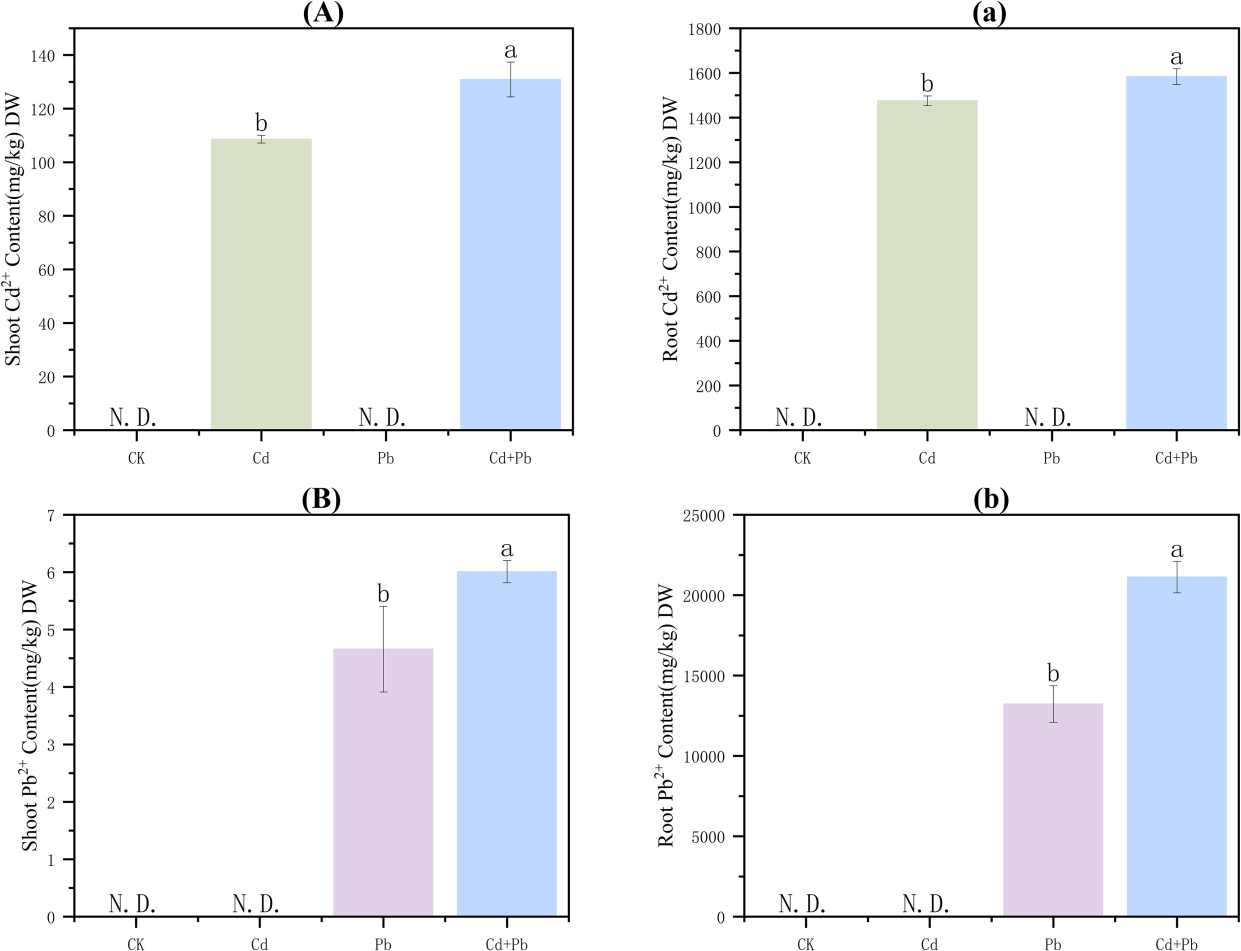
Effects of Cd, Pb and their combined stress on Cd and Pb contents in *A. rosea.* Capital letters **(A, B)** represent the Cd and Pb contents in the shoots, and lowercase letters **(a, b)** represent the Cd and Pb contents in the roots. N.D. indicates that the content is below the lower limit of detection, and DW indicates dry weight. Values are means ± SD (n = 3). Values with a different letter within a sampling date are significantly different (*p* < 0.05).

No Cd or Pb was detected in CK plants. Both metals primarily accumulated in the roots of *A. rosea*. Relative to the Cd group, Cd content in shoots and roots of the Cd + Pb group increased by 20.6% and 7.4%, respectively (*p<* 0.05). Compared with the Pb group, in the Cd + Pb group, the content of Pb in the shoots increased substantially by 29.0% (*p<* 0.05), and by 59.7% in the roots (*p<* 0.05) (**Figure 6**).

### 3.7 Subcellular distribution of Cd and Pb

**Figure 7:**
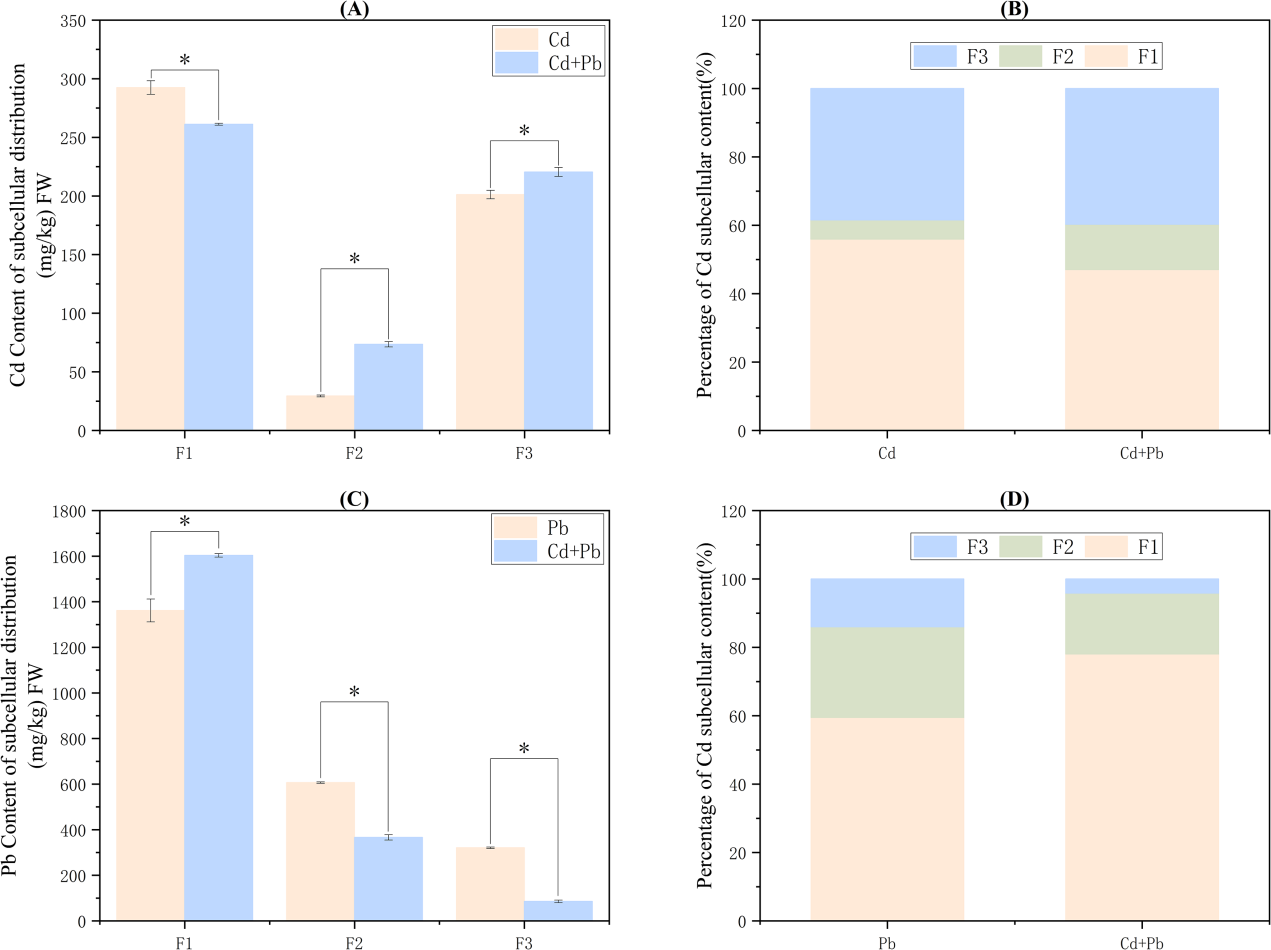
Effects of cadmium, lead, and their combined stress on the contents of cadmium and lead in the root subcellular fractions of *A. rosea*. **(A)** shows the Cd content in the root subcellular fractions of *A. rosea*; **(B)** shows the proportion of Cd in the root subcellular fractions of *A. rosea*; **(C)** shows the Pb content in the root subcellular fractions of *A. rosea*; **(D)** shows the proportion of Pb in the root subcellular fractions of *A. rosea*. FW indicates fresh weight. F1: cell wall component; F2: organelle component; F3: soluble component; * indicates a significant difference between treatments (*p* < 0.05).

Cd primarily accumulated in the cell wall fraction (F1), while Pb primarily accumulated in the cell wall fraction (F1) and soluble fraction (F3). In comparison to the Cd group, the Cd content in the cell wall fraction (F1) markedly decreased by 10.5% in the Cd + Pb group (*p<* 0.05), while the contents in the organelle fraction (F2) and soluble fraction (F3) increased substantially by 150.0% and 9.6% (*p<* 0.05), respectively (**Figures 7A, B**). For Pb, the Cd + Pb treatment increased F1 content by 17.7% (*p<* 0.05), while the organelle fraction (F2) and soluble fraction (F3) contents decreased significantly by 39.6% and 73.4% (*p<* 0.05), respectively (**Figures 7C, D**).

### 3.8 Chemical forms of Cd and Pb

**Figure 8:**
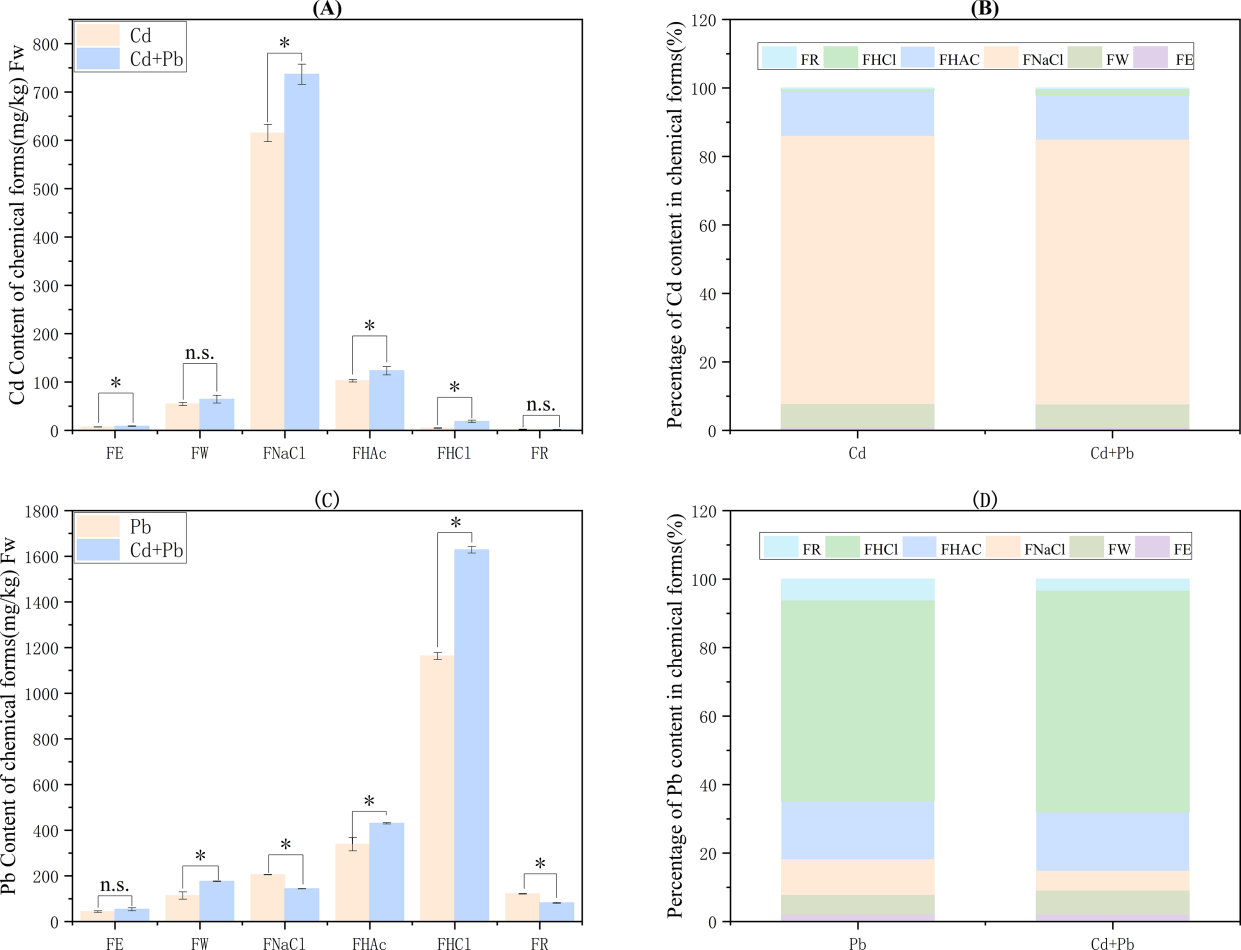
Effects of Cd, Pb and their combined stress on the contents of Cd and Pb in various chemical forms in the roots of *A. rosea.* **(A)** shows the contents of Cd in various chemical forms in the roots of *A. rosea*; **(B)** shows the proportions of Cd in various chemical forms in the roots of *A. rosea*; **(C)** shows the contents of Pb in various chemical forms in the roots of *A. rosea*; **(D)** shows the proportions of Pb in various chemical forms in the roots of *A. rosea*. Fw indicates fresh weight. FE is an 80% ethanol extract; FW is a double-distilled aqueous extract; FNaC1 is l mol/L NaC1 extract; FHAc is a 2% glacial acetic acid extract; FHCI is a 0.6 mol/L hydrochloric acid extract; FR stands for the residual form. * indicates a significant difference between treatments (*p* < 0.05), and n.s. indicates no significant difference between treatments.

In the Cd + Pb group, Cd contents in FE, FNaCl, FHAc, and FHCl forms increased significantly by 23.1%, 19.8%, 20.1%, and 279.5% compared with the Cd group (*p<* 0.05), respectively. There was no significant difference in the contents of Cd in FW and FR forms (*p* > 0.05) (**Figures 8A, B**). FHCl and FHAc are the primary chemical forms of Pb in the roots. The contents of Pb in the FW, FHAc, and FHCl forms increased significantly by 55.1%, 27.1%, and 40.0% (*p<* 0.05), respectively. The contents of Pb in the FNaCl and FR forms decreased significantly by 30.1% and 32.5% (*p<* 0.05), respectively. There was no significant change in the content of Pb in the FE form (*p* > 0.05) (**Figures 8C, D**).

### 3.9 Observation of the root ultrastructure

**Figure 9:**
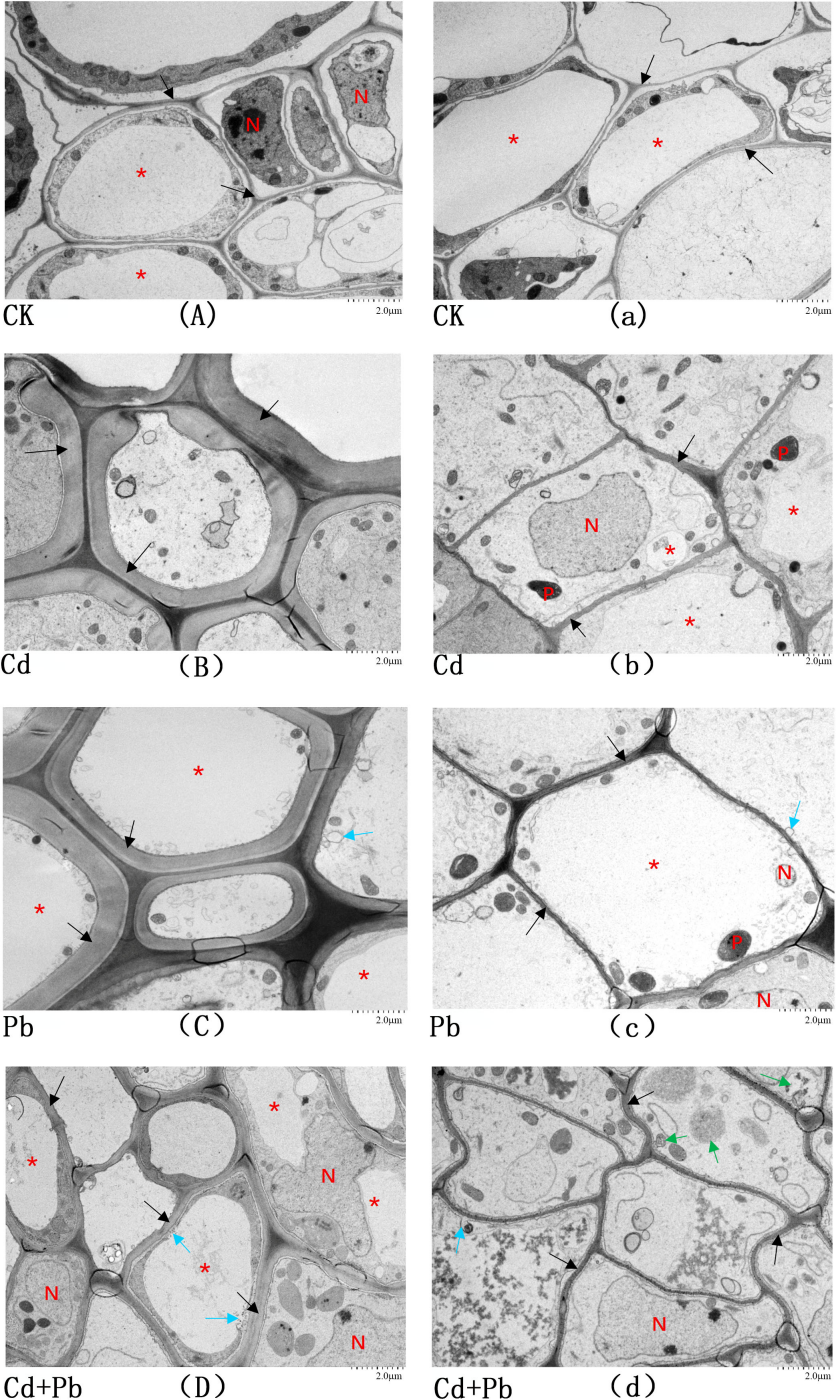
Ultrastructure of root cells of *A. rosea* under Cd, Pb and their combined stress. Capital letters **(A–D)** represent the cell structure in the center of the vascular bundle of *A. rosea* root system under an electron microscope at 4000x magnification, while lowercase letters **(a–d)** represent the cell structure around the vascular bundle of *A. rosea* root system under an electron microscope at 4000x magnification. N: nucleus; P: plastid; *: Vacuole; Black ↑: cell wall; Light blue ↑: deformed cell membrane; Green ↑: Autophagosomes.

The ultrastructural characteristics of root cells under each treatment are presented in **Figure 9**. In the CK samples, all fundamental cellular components were present, and the cellular structures were relatively intact (**Figure 9A, a**). Similarly, in the Cd−treated roots, the basic ultrastructural elements were preserved. The nuclear envelope was intact, and there was less heterochromatin (**Figure 9B, b**). The vacuole edges were relatively smooth with clear boundaries. There were no abnormalities in the structures. The nuclear envelope was intact in the Pb group; the chromatin distribution was largely normal, and there was slight local deformation of the cell membrane and detachment between the membrane and the wall (**Figure 9C, c**). However, this phenomenon was not observed in the control group (**Figure 9A, a**). In the Cd + Pb group, the cell structures were relatively intact, but local damage or deformation of the cell membrane was observed (**Figure 9D, d**). Distinct separation between the plasma membrane and cell wall was observed (**Figure 9D, d**), and the boundaries of some vacuoles were unclear (**Figure 9d**). However, in the CK group, the cell wall was tightly attached to the cell membrane, and the tonoplast was clear (**Figure 9A, a**). These observations indicate that combined Cd and Pb stress exerts pronounced cytotoxic effects on the root cell structure of *A. rosea* and damages the membrane system of root cells.

### 3.10 Correlation analysis

**Figure 10:**
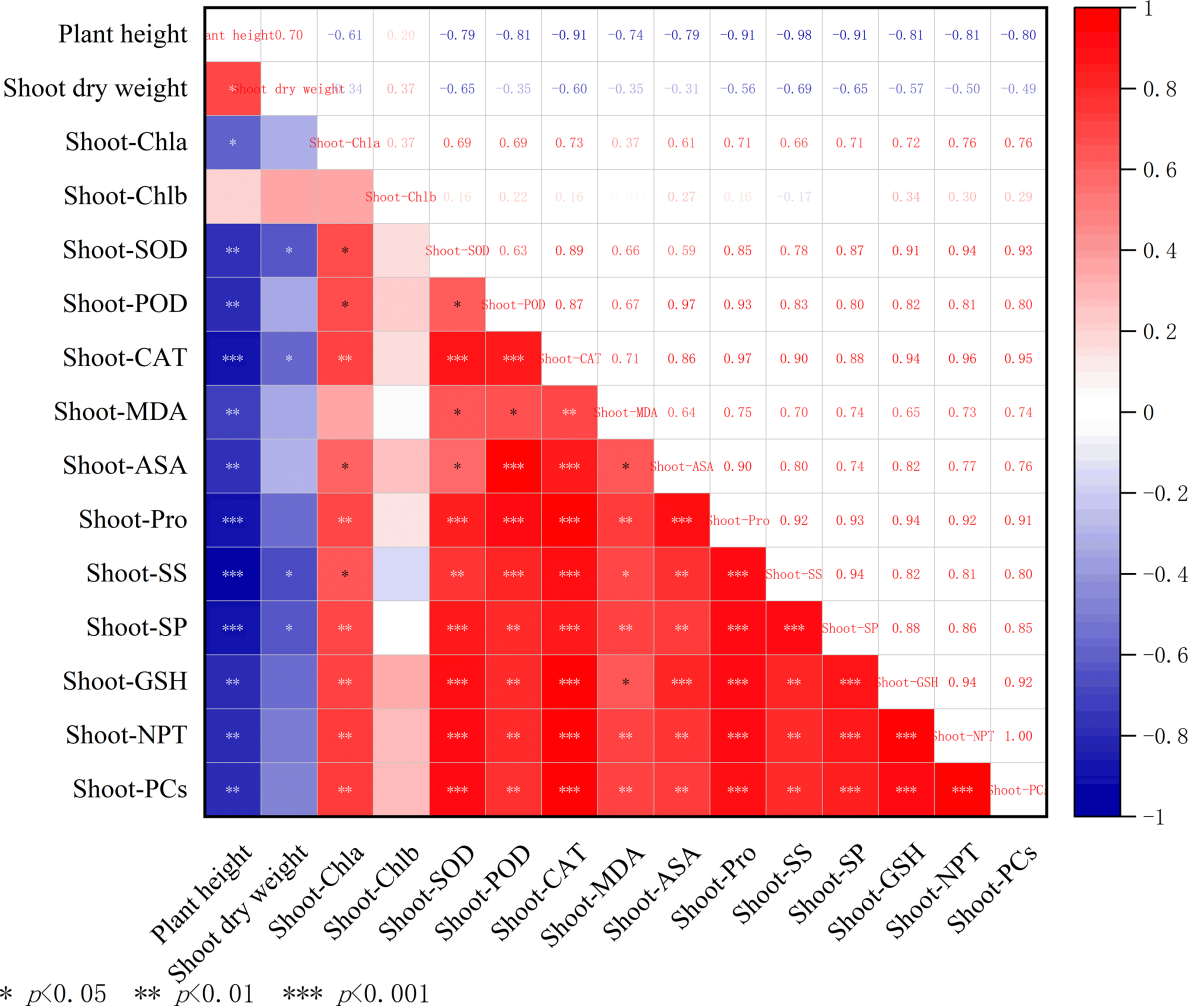
Correlation analysis of various indices in the shoots of *A. rosea* under Cd, Pb, and their combined stress. *, ** and *** indicate significant differences at 5%, 1% and 0.1% levels, respectively.

As shown in **Figure 10**, there were significant correlations among the various indices of *A. rosea* shoot tissues under Cd and Pb stress. Pant height was extremely significantly negatively correlated (*p<* 0.01) with the activities of antioxidant enzymes (SOD, POD, and CAT) and osmotic adjustment substances (Pro and SS), while it was positively correlated with chlorophyll content. Notably, the three antioxidant enzymes (SOD, POD, and CAT) had a high degree of synergy (*p<* 0.01) and were significantly positively correlated with the content of MDA. The contents of Pro, SS, and GSH were highly related (*p* < 0.01), whereas their correlations with PCs were comparatively weak.

**Figure 11:**
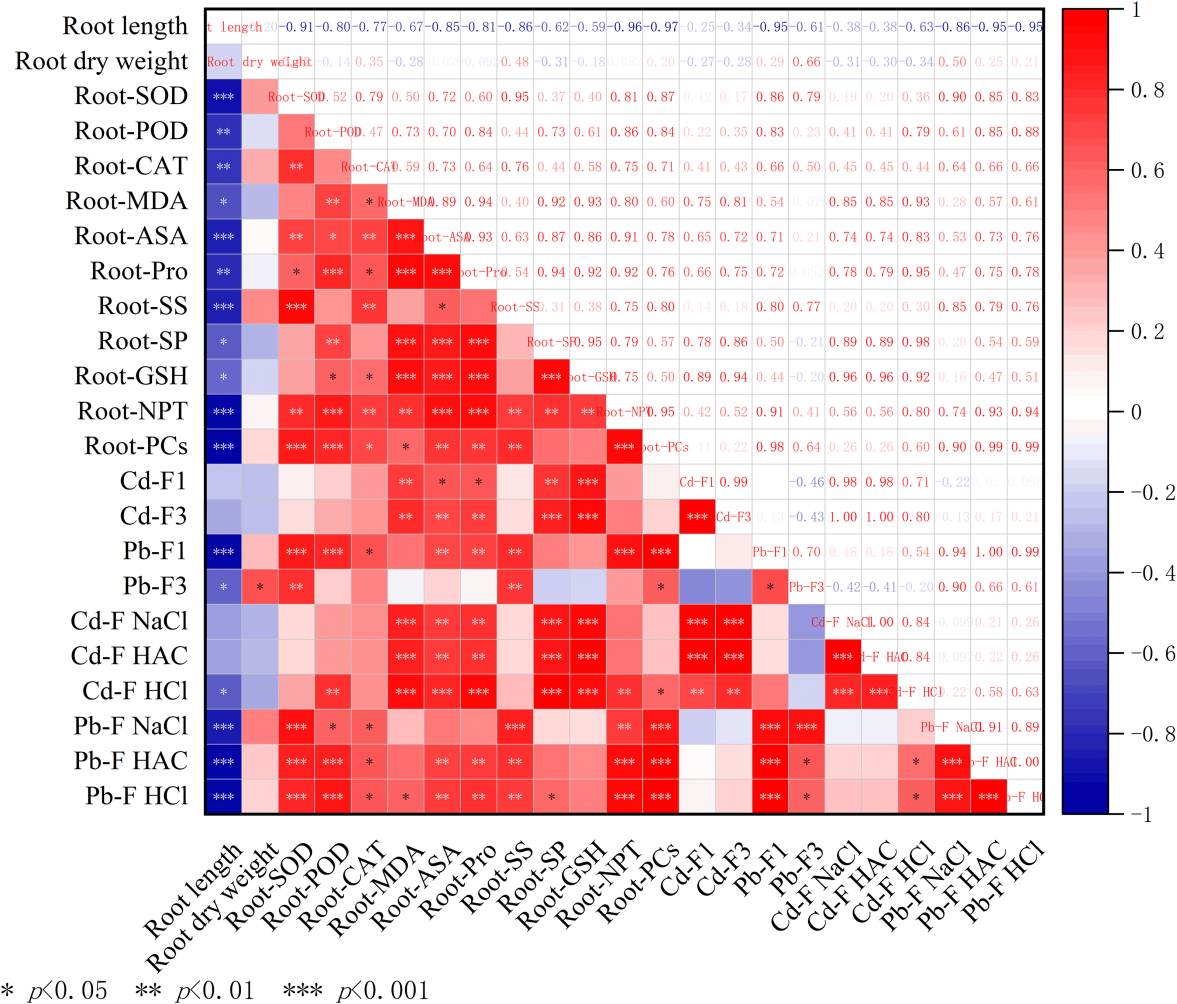
Correlation analysis of various indices in the roots of *A. rosea* under Cd, Pb, and their combined stress. *, ** and *** indicate significant differences at 5%, 1% and 0.1% levels, respectively.

As shown in **Figure 11**, the root indices exhibited more complex correlation patterns under Cd and Pb stress. The root length was significantly positively correlated (*p* < 0.01) with the root dry weight and all physiological indices, suggesting a coordinated relationship between root growth and physiological responses. However, indices, such as the subcellular distribution and chemical forms of Cd and Pb, were significantly positively correlated (*p* < 0.05) with the activities of antioxidant enzymes (SOD, POD, and CAT) and the osmotic adjustment system. The osmotic adjustment compounds, such as Pro and SS, were highly positively correlated with the content of MDA (*p* < 0.01). In addition, PCs significantly positively correlated with the accumulation of Cd and Pb (*p* < 0.01).

### 3.11 Principal component analysis

**Figure 12:**
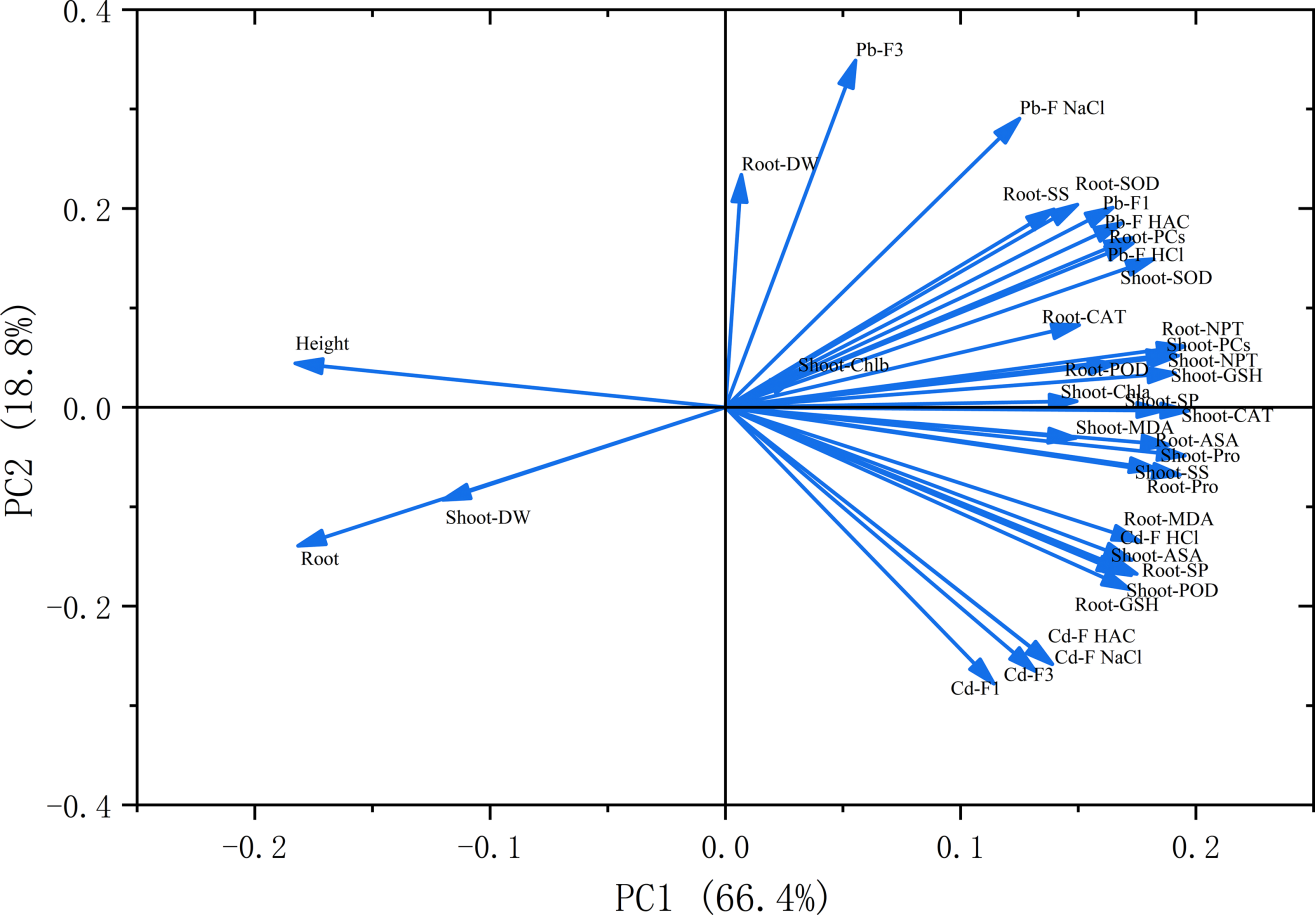
Principal component analysis of various indices in the roots of *A. rosea* under Cd, Pb and their combined stress.

Given the strong correlations among numerous indices, PCA was employed to reduce dimensionality and identify major contributing variables (**Figure 12**). Based on the eigenvalues and contribution rates, the first two principal components were selected from the data of *A. rosea* under different treatments and explained 85.2% of the total variance (exceeding the cumulative threshold of 85%). Most of the traits on PC1 had high positive loadings (close to or exceeding 0.19), while plant height and root length had low negative loadings (−0.185 and −0.196, respectively). PC2 had a more complex mix of positive and negative loadings. The shoot DW, root-MDA, root-Pro, root-SP, root-GSH, and several heavy metal forms had high positive loadings (> 0.22). In contrast, the root DW, shoot-SOD, Pb-F3, and others indices had high negative loadings (<0.24). The PCA results showed that most physiological traits responded positively to metal stress, while plant height and root length were strongly inhibited. This indicates that *A. rosea* activates a suite of defensive mechanisms to mitigate Cd and Pb toxicity.

## 4 Discussion

Heavy metal stress exerts its effects on plants primarily through alterations in growth and development. Therefore, morphological growth indicators can be used to evaluate plant responses to metal toxicity (Jaishankar et al., 2014). In this study, Cd, Pb, and their combined stress were toxic to *A. rosea* seedlings and inhibited their growth. This included a reduction in plant height, root length, and biomass. Among them, the combined stress of Cd + Pb had the most severe inhibitory effect on *A. rosea* growth. The content of chlorophyll is the most intuitive indicator of the photosynthetic capacity of plants. It has been found that heavy metals impair photosynthesis by damaging the structures of chloroplasts, inhibiting the absorption of light energy, disrupting the electron transport chains, and reducing the activity of enzymes related to the Calvin cycle (Hoque et al., 2021). Sun et al. (2019) found that the contents of chlorophyll a and total chlorophyll in spreading diamond flower (*Scleromitrion diffusum* R. J. Wang) increased under Cd, Pb, and their combination, consistent with the results of this study. In the present study, chlorophyll a and total chlorophyll increased significantly under all treatments, whereas chlorophyll b increased under Cd and Pb alone but not under combined stress. Chlorophyll b appears more vulnerable to Cd and Pb toxicity, probably because heavy metal stress suppresses its biosynthetic enzymes (Sumalan et al., 2023). The results indicate that Cd, Pb, and their combined stress can promote the accumulation of chlorophyll in *A. rosea*, with the combined treatment showing the strongest effect.

Heavy metal stress disrupts the dynamic balance of ROS in plants, thereby inducing oxidative stress responses. This leads to the massive production of ROS, which exacerbates the peroxidation of lipids in cell membranes and causes membrane damage (Mansoor et al., 2023). SOD, POD, and CAT are the primary antioxidant enzymes in plants. MDA is a product of membrane lipid peroxidation, and ASA is an important antioxidant. In this study, Cd, Pb, and Cd + Pb significantly increased SOD, POD, and CAT activities, as well as MDA and ASA contents. Duan et al. (2022) also found that the activities of SOD, POD, and CAT in *A. rosea* increased under individual Pb stress. In a study on Cd stress in potato (*Solanum tuberosum* L.), plants enhanced their antioxidant performance by increasing the activities of SOD, POD, and CAT (Li et al., 2019). Under Cd stress, the content of MDA in watermelon (*Citrullus lanatus* Matsum. & Nakai) (Khan et al., 2021), pepper (*Capsicum annuum* L.) (Kaya, 2021), and maize (*Zea mays* L.) (Zhang et al., 2017) seedlings increased significantly. These results indicate that *A. rosea* mitigates membrane lipid peroxidation and protects its membrane structures by increasing the activities of SOD, POD, and CAT, as well as the ASA content under Cd and Pb stress. However, combined Cd + Pb stress induces more severe oxidative injury in *A. rosea*.

Proline accumulation is widely recognized as an indicator of plant resistance (Ghosh et al., 2022). Under stress conditions, the decomposition of starch and other sugars leads to an increase in the contents of SS. The products of photosynthesis are directly converted into low−molecular−weight osmolytes. The osmoregulatory compounds can regulate the osmotic potential of plant cells, thereby maintaining water content homeostasis. Treatment of yellow iris (*Iris pseudacorus* L.) with Cd, Pb, and their stress combination significantly increased the contents of Pro, SS, and SP (Zhou et al., 2012), consistent with the findings of this study. This indicates that *A. rosea* experienced osmotic stress, which was more severe when the plants were subjected to combined stress. The plant maintains its cellular osmotic balance by increasing osmotic regulatory compound contents.

GSH is the most abundant low-molecular-weight thiol compound in cells. It can directly scavenge ROS in plants and serves as a precursor for the biosynthesis of PCs (Verbruggen et al., 2009). In addition, NPT and the PCs are key high-affinity groups that take part in the detoxification process of plant cells. They play an indispensable role in the detoxification of heavy metals and the maintenance of intracellular homeostasis (Rabêlo et al., 2018). When the plants are subjected to stress, the levels of GSH, NPT, and PCs in the plants increase as part of their detoxification response (Kaya et al., 2021). In this study, compared with the CK group, the contents of GSH, NPT, and PCs in the shoots and the roots of the Cd, Pb, and Cd + Pb groups increased significantly. Moreover, the contents of GSH, NPT, and PCs under the Cd + Pb stress were also significantly higher than those under the single Cd or Pb stress. This is consistent with the conclusions of Kaya et al. (2021). GSH content in patchouli (*Pogostemon cablin* Benth.) increased by 94% under Cd stress (Fu et al., 2025). GSH promotes the formation of Cd-GSH complexes by activating members of the GST gene family in rice (*Oryza sativa* L.) subjected to Cd and arsenic (As) stress, facilitating the sequestration of heavy metals into vacuoles for detoxification (Wang et al., 2025). These results indicate that *A. rosea* enhances its tolerance to Cd and Pb by synthesizing greater quantities of cellular chelators, enabling more effective binding and detoxification of metal ions.

Cd and Pb are non-essential elements for plant growth, and their accumulation in plants can result in substantial toxicity. The cell wall of plant roots acts as the initial barrier for the absorption of cations and plays a crucial role in the accumulation of heavy metals (Loix et al., 2017). When the heavy metals on the cell wall reach saturation, some plants can transport heavy metal ions from the cell wall to inactive compartments, such as the vacuoles, thereby reducing damage to plant cells. Thus, plants with strong heavy−metal tolerance often exhibit pronounced compartmentalization mechanisms (Gupta et al., 2013). Moreover, the toxicity and mobility of heavy metals are closely related to their chemical forms in plant cells (Chai et al., 2018). In this study, the absorption of Cd and Pb by *A. rosea* increased under combined Cd and Pb stress. This might be because the coexistence of multiple heavy metals can inhibit the transformation of a single heavy metal from the available form to the unavailable form, thereby increasing its bioavailability. Consequently, the combined pollution of heavy metals is more toxic (Zhao et al., 2023). Studies by Liu et al. (2008). have also shown that *A. rosea* can effectively accumulate Cd and Pb under Cd and Pb combination. Studies on common tobacco (*Nicotiana tabacum* L.) have also shown that combined Cd and Pb stress can enhance Cd and Pb absorption (Wang et al., 2023). Additionally, combined Cd and Pb stress increased the content of Cd in the soluble fractions and Pb in the cell wall fractions. This indicated that cell wall immobilization and vacuolar compartmentalization are the primary mechanisms underlying the ability of *A. rosea* to tolerate Cd and Pb. This pattern is consistent with observations in the hyperaccumulator Asian common ladyfern (*Athyrium yokoscense* Franch. & Sav.) (Nishizono et al., 1987). Under the combined stress of Cd and Pb, both the contents of Cd in the FE, FNaCl, FHAc, and FHCl forms, and the contents of Pb in the FW, FHAc, and FHCl forms increased significantly. Additionally, the toxicity and mobility of Cd and Pb were enhanced, and there was a synergistic toxic effect between Cd and Pb. This is consistent with the findings in *Crassocephalum crepidioides* S. Moore and *Ageratum conyzoides* L (Zhu et al., 2017).

Excessive Cd and Pb in the environment can damage the antioxidant system of plants, induce excessive ROS production, cause membrane lipid peroxidation, and damage cellular structures (Garnier et al., 2006; Iannone et al., 2010). In this study, Cd exposure reduced heterochromatin in the root cells of *A. rosea*. This may be due to the increased content of calcium ions, which leads to a decrease in nuclear cellulose. Consequently, such changes in heterochromatin result in damage to the structure and function of the cell nucleus (Hao et al., 2016). Pb exposure caused deformation of the plasma membrane and separation between the membrane and cell wall. Under Cd + Pb stress, membrane deformation and membrane–wall detachment were also observed, with unclear boundaries around some vacuoles. The detachment of the cell wall may be attributed to an induction of an excessive accumulation of lignin and callose in the cell wall owing to the Pb ions, which reduces its plasticity and thus leads to the detachment between the cell membrane and the cell wall (Hu et al., 2009). Moreover, studies on rice (Wang et al., 2014) and Amur silver grass (*Miscanthus sacchariflorus* Benth. & Hook. f. ex Franch.) (Xin et al., 2018) have also demonstrated the toxic effects of Cd and Pb on the ultrastructure of cells.

Heavy metal tolerance in plants is a complex trait influenced by multiple physiological and biochemical factors. Therefore, when evaluating the heavy metal tolerance of plants, a comprehensive assessment using multi-aspect indicators should be adopted to avoid the limitations of single−parameter assessments (Li et al., 2020; Wang et al., 2017). Correlation analysis revealed significant positive correlations among the physiological indices of *A. rosea* subjected to Cd, Pb, and their combined stress, and similar results were also observed in plants of the Chenopodiaceae family under a combination of heavy metal pollutants (Liu et al., 2021). PCA identified three principal components that effectively summarized the variation among all indicators. The results indicated that most traits responded positively to metal stress, while plant height and root length were suppressed, demonstrating that *A. rosea* employs multiple defensive strategies to mitigate cadmium and lead stress. PCA also addressed multicollinearity and increased the reliability of this evaluation. Notably, a hydroponic culture system was adopted in this study to ensure precise control of experimental variables. In actual soil environments, numerous factors need to be considered, including pH, electrical conductivity, and organic matter content, all of which can influence heavy metal accumulation in plants. Future research should therefore incorporate soil−based experiments to evaluate the practical phytoremediation potential of *A. rosea*.

## 5 Conclusions

This study explored the physiological responses and characteristics of the tolerance of *A. rosea* under Cd, Pb, and combined Cd + Pb stress. All heavy metal treatments inhibited the growth of *A. rosea* in terms of plant height, root length, biomass, and other aspects, and the inhibition was more severe under combined Cd + Pb stress. The responses of *A. rosea* seedlings to the combination of Cd and Pb stress manifested as increased activities of SOD, POD, and CAT, and increased contents of ASA, SS, SP, Pro, NPT, GSH, and PCs. Cd and Pb also induced ultrastructural alterations in root cells. The accumulation of Cd and Pb by the roots is one of the mechanisms used by *A. rosea* to tolerate these pollutants. In addition, *A. rosea* enhances its tolerance to Cd and Pb by accumulating Cd and Pb in inactive parts, such as the cell walls and vacuoles, and transforming Cd and Pb into chemical forms with lower toxicity. These results elucidate the physiological responses and tolerance of plants under combined Cd and Pb stress and provide possible strategies to improve the resilience of plants in environments polluted by both Cd and Pb. This study used hydroponic culture to investigate the growth, physiological responses, and tolerance characteristics of *A. rosea* to Cd and Pb, but no molecular biological studies were performed. Future research should pursue two directions: on the one hand, field experiments should be carried out to evaluate the practical phytoremediation performance of *A. rosea*; on the other hand, molecular studies should be undertaken to elucidate the regulatory pathways underlying its tolerance to Cd and Pb.

## Data Availability

The original contributions presented in the study are included in the article/**Supplementary Material**. Further inquiries can be directed to the corresponding author.
