# Supplementary material for: Alcea rosea L. responses to Cd and Pb stress: phenotypic, physiological, subcellular, chemical speciation, and ultrastructural analyses
Source: Front Plant Sci. 2026 Mar 20;17:1778764. doi: 10.3389/fpls.2026.1778764 (PMC13046533; doi:10.3389/fpls.2026.1778764)
Supplement: Supplementary file 1 [file Table1.docx]

**Table S 1** Modified Hoagland hydroponic nutrient solution formula

| Compound name | Standard concentration (mg/L) | 1/8 strength concentration (mg/L) |
| --- | --- | --- |
| Ca(NO_3_)_2_ | 945 | 118.125 |
| K_2_SO_4_ | 607 | 75.875 |
| NH_4_H_2_PO_4_ | 115 | 14.375 |
| MgSO_4_ | 493 | 61.625 |
| EDTA | 20 | 2.500 |
| FeSO_4_ | 15 | 1.875 |
| H_3_BO_3_ | 2.86 | 0.358 |
| Na_2_B_4_O_7_·10H_2_O | 4.5 | 0.563 |
| MnSO_4_ | 2.13 | 0.266 |
| CuSO₄ | 0.05 | 0.006 |
| ZnSO_4_ | 0.22 | 0.028 |
| (NH_4_)_2_SO_4_ | 0.02 | 0.003 |
